# Complete genome sequence of *Eubacterium brachy* GTC20104 isolated from a human pleural effusion

**DOI:** 10.1128/mra.00747-25

**Published:** 2025-09-12

**Authors:** Jun Yonetamari, Masahiro Hayashi, Yoshinori Muto, Kaori Tanaka

**Affiliations:** 1 United Graduate School of Drug Discovery and Medical Information Sciences, Gifu University 520344 , Gifu City, Gifu, Japan; 2 Division of Clinical Laboratory, Gifu University Hospital 476117, Gifu, Japan; 3 Division of Anaerobe Research, Life Science Research Center, Gifu University 201111, Gifu City, Gifu, Japan; 4 Institute for Glyco-core Research iGCORE, Gifu University 12785, Gifu City, Gifu, Japan; 5 Gifu University Center for Conservation of Microbial Genetic Resource, Gifu, Japan; 6 Center for One Medicine Innovative Translational Research (COMIT), Gifu University 12785, Gifu, Japan; Nanchang University, Nanchang, Jiangxi, China

**Keywords:** *Eubacterium*, anaerobe, genome

## Abstract

The genus *Eubacterium* comprises gram-positive anaerobic bacteria. It has long been recognized as a component of human oral and gut microbiota. Here, we report the complete genome sequence of a novel strain, *Eubacterium brachy* GTC 20104, isolated from a human pleural effusion, consisting of a 1,593,027-bp circular chromosome.

## ANNOUNCEMENT

*Eubacterium brachy* is an anaerobic coccobacillus frequently isolated from human oral lesions (1). Its pathogenic potential has been suggested by its high prevalence of 35–42% in subgingival periodontitis samples (1). *E. brachy* has been reported in other body sites, including empyema fluid (2). Strain GTC20104 was isolated from a human pleural effusion sample in 2020. Isolation and maintenance were performed using Brucella HK agar plates (Kyokuto Pharmaceutical Industrial, Tokyo, Japan) supplemented with 5% sheep blood and incubated at 37°C for 48 h under anaerobic conditions (82% N_2_, 10% CO_2_, and 8% H_2_). This study was conducted following the principles of the Declaration of Helsinki. Identification using matrix-assisted laser desorption/ionization time-of-flight mass spectrometry (Bruker, Bremen, Germany) was unsuccessful.

The strain was subcultured under the same conditions as the initial isolation, and a single colony was cultured in Brucella broth for DNA extraction. Genomic DNA was extracted from the resulting cell pellet using Quick Taq HS DyeMix (TOYOBO Co., Ltd., Osaka, Japan). The entire isolate genome was sequenced using both long- (Oxford Nanopore Technologies, Tokyo, Japan) and short-read sequencing (Illumina Inc., San Diego, CA, USA) (3–5). For long-read sequencing, a library was prepared using a ligation sequencing kit (SQK-NBD114-24; Oxford Nanopore Technologies) without DNA shearing. The sequencing run generated 4,951,692 raw reads, with an N50 read length of 4,877 bp (Table 1). Small DNA fragments were removed (Short Read Eliminator XS; Circulomics, Baltimore, MD, USA), and sequencing was performed using a PromethION 2i instrument with a FLO-PRO114M flow cell. Long-read data were base-called in super-accuracy mode using the default base-calling pipeline in MinKNOW version 23.11.8 (Oxford Nanopore Technologies). The raw long reads were trimmed and quality-filtered using NanoFilt v.2.7.1 (6) with the parameters “-q 10 –headcrop 50 -l 1000.” An Illumina DNA Prep (M) Tagmentation Kit (Illumina) was used for library construction for short-read sequencing, followed by 2 × 151 bp paired-end sequencing on a NovaSeq 6000 platform (Illumina). Raw short reads were processed using fastp v.0.20.1 (3) with “-q 30 -n 20 -t 1 -T 1” parameters. Short-read quality was assessed using fastp, and mean long-read quality was assessed using NanoPlot v.1.32.1 (6). High-quality short reads (>87% bases >Q30 on average) and long reads (mean read quality of 16.7) were assembled using Unicycler v.0.4.8 with the default settings (7). Assembly circularization and rotation were performed automatically using the Unicycler to start with dnaA on the forward strand. The assembled contig graph was visualized and assessed for integrity using BlobTools v.1.0. Genome annotation was performed using DFAST (DDBJ Fast Annotation and Submission Tool) with default settings to predict coding sequences, rRNAs, tRNAs, and clustered regularly interspaced short palindromic repeats (CRISPR) elements.

**TABLE 1:** Information regarding the complete genome sequence of *Eubacterium brachy* GTC20104, isolated from a human pleural effusion

Parameter	Strain name
	*Eubacterium brachy* GTC20104
Illumina sequencing^*a*^
No. of reads	4,951,692
Size (kb)	740,355
Avg coverage (x)	465
DRA accession no.	DRX640103
ONT sequencing^*a*^
No. of reads	395,990
Size (kb)	1,336,347
Average read length (bp)	3,374.7
Average coverage (x)	839
N50	4,877
DRA accession no.	DRX640102
Assembly
Estimated genome completeness (%)^*b*^	100.0
Estimated genome contamination (%)^*b*^	0.0
Genome structure	One chromosome
DDBJ/GenBank accession no.	AP040261
Genome size (bp)	1,593,027
GC content (%) (chromosome/ plasmid name)	38.0
No. of coding sequences^*c*^	1,399
Number of rRNAs^*c*^	6
Number of tRNAs^*c*^	37
Number of CRISPRs^*c*^	2

^*a*^ DRA, DNA DataBank of Japan (DDBJ) sequence read archive.

^*b*^ Determined using CheckM.

^*c*^ DFAST, DDBJ fast annotation and submission tool.

The taxonomic identification of strain GTC20104 was performed using the Type Strain Genome Server TYGS (8). Genome- and rRNA-based comparisons supported *E. brachy* ATCC 33089 as the most closely related strain. The digital DNA–DNA hybridization value (88.6%) exceeded the species delineation threshold, supporting the classification of strain GTC20104 as *E. brachy*. This whole-genome sequence adds valuable insight to the limited genomic data available for the genus *Eubacterium*.

## Data Availability

The complete genome sequence of Eubacterium sp. GTC20104, isolated from human pleural effusion, was deposited in the DNA Data Bank of Japan (DDBJ; https://www.ddbj.nig.ac.jp/index.html) under the accession number AP040261. Raw sequence data for GTC20104 have been deposited in the DDBJ Sequence Read Archive under accession numbers DRX640102 and DRX640103.
